# Indoleamine 2, 3-dioxygenase (IDO) increases during renal fibrogenesis and its inhibition potentiates TGF-β 1-induced epithelial to mesenchymal transition

**DOI:** 10.1186/s12882-017-0702-7

**Published:** 2017-09-06

**Authors:** Luiz Henrique Gomes Matheus, Gislene Mendes Simão, Taíssa Altieri Amaral, Rodrigo Barbosa Oliveira Brito, Camila Soares Malta, Yves Silva Teles Matos, Alexandre Chagas Santana, Gabriela Gomes Cardoso Rodrigues, Maria Clara Albejante, Erna Elisabeth Bach, Maria Aparecida Dalboni, Cleber Pinto Camacho, Humberto Dellê

**Affiliations:** Postgraduate Program in Medicine, Universidade Nove de Julho (UNINOVE), Rua Vergueiro, 235, 2º subsolo, São Paulo, 01504-001 São Paulo Brazil

**Keywords:** Indoleamine 2, 3-dioxygenase, 1-methyl-D-tryptophan, Renal fibrosis, Epithelial to mesenchymal transition, TGF-β 1, Renal inflammation

## Abstract

**Background:**

Indoleamine 2, 3-dioxygenase (IDO) is an immunomodulatory molecule that has been implicated in several biological processes. Although IDO has been linked with some renal diseases, its role in renal fibrosis is still unclear. Because IDO may be modulated by TGF-β1, a potent fibrogenic molecule, we hypothesized that IDO could be involved in renal fibrosis, especially acting in the TGF-β1-induced tubular epithelial-mesenchymal transition (EMT). We analyzed the IDO expression and activity in a model of renal fibrogenesis, and the effect of the IDO inhibitor 1-methyl-tryptophan (MT) on TGF-β1-induced EMT using tubular cell culture.

**Methods:**

Male Wistar rats where submited to 7 days of UUO. Non-obstructed kidneys (CL) and kidneys from SHAM rats were used as controls. Masson’s Tricrome and macrophages counting were used to chatacterize the tissue fibrosis. The EMT was analysed though immunohistochemistry and qRT-PCR. Immunohistochemestry in tissue has used to show IDO expression.

MDCK cells were incubated with TGF- β1 to analyse IDO expression. Additionally, effects of TGF- β1 and the inhibition of IDO over the EMT process was acessed by immunoessays and scrath wound essay.

**Results:**

IDO was markedly expressed in cortical and medular tubules of the UUO kidneys. Similarly to the immunolocalizaton of TGF- β1, accompanied by loss of e-cadherin expression and an increase of mesenchymal markers. Results in vitro with MDCK cells, showed that IDO was increased after stimulus with TGF-β1, and treatment with MT potentiated its expression. MDCK stimulated with TGF-β1 had higher migratory activity (scratch-wound assay), which was exacerbated by MT treatment.

**Conclusions:**

IDO is constitutively expressed in tubular cells and increases during renal fibrogenesis. Although IDO is induced by TGF-β1 in tubular cells, its chemical inhibitor acts as a profibrotic agent.

## Background

Chronic kidney disease (CKD) is a worldwide growing public health problem. The CKD prevalence in the US doubled in the last decade, and in Brazil, where approximately 90,000 patients are currently undergoing dialysis, this prevalence is also rapidly growing [1]. The pathogenesis of CKD involves a complex mechanism with hemodynamic and inflammatory factors that culminate in glomerulosclerosis and tubulointerstitial fibrosis. The renal fibrosis is strongly induced by transforming growth factor-β 1 (TGF-β 1), especially by activation of fibroblasts (myofibroblasts) and production of extracellular matrix components [2].

The origin of fibrosis-forming kidney myofibloblasts has long been a subject of debate. In addition to the traditional sources of myofibroblast such as from pericytes [3], from interstitial fibroblasts and from bone marrow cells [4], injured epithelial cells have been thought to directly contribute to the myofibroblast pool by the process of epithelial-to-mesenchymal transition (EMT).

In the EMT process, a polarized epithelial cell assumes a mesenchymal phenotype, leading to disruption of the epithelial layers via degradation of the basement membrane, invasiveness, and production of extracellular-matrix-degrading enzymes [5–7]. The tubular cells lose epithelial markers and increase the production of mesenchymal proteins. Studies have shown that renal tubular cells express low levels of α-smooth muscle actin (α-SMA), a traditional mesenchymal marker, which is strongly increased by TGF-β1 [8, 9]. Induction of EMT is likely to be a centrally important mechanism for the progression of CKD. The blockage of the EMT with hepatocyte growth factor prevents the renal interstitial fibrosis [10].

Indoleamine 2, 3-dioxygenase (IDO) is an enzyme that has been linked with several disorders mediated by TGF-β 1, acting as an important mediator or as an efficient marker. IDO is induced by TGF-β 1 in some cell types, forming a mechanism recognized as TGF-β-IDO axis in dendritic cells [11].

Since Munn et al., described the role of IDO during pregnancy, protecting the embryos against the maternal immune system, IDO has been pointed as an immunomodulatory molecule [12]. During an inflammatory response, IDO catalyzes the first and rate-limiting step in tryptophan catabolism, leading to an increase of kynurenine catabolites, which act as local immunosuppressive agents [13]. Because IDO has immunomodulatory proprieties, it is strongly associated with kidney allograft survival [14, 15], demonstrating a protective effect for tubular cells [16, 17]. Interestingly, IDO has been found in other types of renal disease. In a model of nephrotoxic serum nephritis, IDO expression was found in glomerular and tubular cells, and its inhibition with 1-methyl-tryptophan intensified the renal injury [18]. On the other hand, induction of IDO expression in tubular cells was associated with increased apoptosis in a model of renal ischemia-reperfusion injury [19]. Additionally, IDO expression was also increased in models of diabetic nephropathy [20], and its increase was correlated with loss of glomerular filtrate rate in patients with CKD [21]. Given the above considerations, there is evidence pointing to a possible role of IDO in renal diseases.

Recently, our group showed that the IDO modulates the TGF-beta1-induced EMT in bladder cancer cells as a possible way to promote metastasis since its chemical inhibition with 1-methyl-tryptophan intensified the markers for EMT [22].

In this study, we analyzed the expression and activity of IDO in a model of renal fibrosis characterized by EMT, and the effect of 1-methyl-tryptophan on TGF- β 1-induced EMT using tubular cell culture.

## Methods

### Animals

Male Wistar rats (approximately 280 g weight) were obtained from an established colony at the Nove de Julho University, São Paulo, Brazil. The animals were housed in standard cages and maintained in a 22 °C room with a 12-h light/dark cycle, and allowed ad libitum access to food and water before and after the unilateral ureteral obstruction (UUO) procedure. All experimental procedures were conducted in accordance with international standards of animal care and experimentation and were approved by the Institutional Research Ethics Committee of the Nove de Julho University, São Paulo, Brazil (protocol AN1/2013).

### UUO model and experimental design

Animals were carefully anesthetized with Ketamin (Ketamin-S, São Paulo, Brazil) and Xylazine (Rompun, Bayer, Leverkusen, Germany) injected intraperitoneally. UUO model was performed as described previously, with minor modification [20].

Briefly, the left kidney and ureter were exposed through a small abdominal incision and ligated using 4–0 silk. During the surgical procedure, animals were appropriately hydrated with physiological saline solution, and their body temperature was kept at approximately 37 °C using an adjustable heating pad. Finally, the skin incision was closed in layers with single interrupted sutures. None of the animals developed signs of systemic infections. Ten rats were divided among two groups: SHAM, rats submitted to surgery but without ureteral obstruction, and UUO, rats submitted to surgery with obstruction of the left ureter. The right contralateral kidneys (CL) of the UUO rats were also used as a control. The groups were followed for 7 days. The kidneys were harvested and one half of each kidney was fixed in Dubosq-Brazil solution for 45 min and, then, post-fixed in buffered 10% formaldehyde solution and two midcoronal sections were embedded in paraffin for histological and immunohistochemistry analysis. The other half was stored at −80 °C for q-PCR assays.

### Renal histology

Three-micrometer paraffin-embedded kidney sections were mounted on slides and submitted to Masson’s trichrome staining. Histopathology features were determined and calculated using the Image-Pro Plus 7.0 software (Media Cybernetics Inc., Silver Spring, USA), permitting the automated analysis of all morphological alterations. Measurements were performed in the tubulointerstitial compartment. The percentage of Masson’s trichrome staining was calculated relatively to the entire field area (percentage area). All morphologic analyzes were carried out in a blinded fashion under ×200 microscopic magnification.

### Immunohistochemistry

Paraffin sections of renal tissue were cut at 4-μm thickness and subjected to microwave irradiation in citrate buffer to enhance antigen retrieval. After blocking steps with 0.3% hydrogen peroxide and non-fat milk, the following antibodies were used as primary antibodies: anti-rat CD68 (MCA341R, Serotec, Oxford, UK), anti-E-cadherin (Ecad, IS059; Dako Co, Denmark), anti-α-smooth muscle actin (αSMA; IS700; Dako Co, Denmark), anti-vimentin (M0725; Dako Co, Denmark), and anti-IDO (MAB5412; Merck Millipore, Billerica, MA). All antibodies were diluted 1:100. To complete the sandwich, sections were incubated with LSAB+ System-HRP reagents (K0690; Dako Co, Denmark). Finally, DAB substrate-chromogen was used to complete the reaction (K346811; Dako Co, Denmark).

We conducted a quantitative analysis of the positive interstitial cells for ED-1, αSMA and vimentin in a blinded fashion under X200 microscopic magnification, expressed as cells per field. The positive and negative tubular cells for αSMA and vimentin were counted under X200 microscopic magnification and the results are expressed as a percentage of positive cells. To analyze E-cadherin and IDO expression, the positive and negative tubules were counted for presenting as a percentage of positive tubules.

### Real-time PCR

Total RNA from kidney tissue was extracted (at the 4 °C using a tissue homogenizer) by guanidinium thiocyanate-chloroform (Invitrogen, Carlsbad, USA), and isolated according to the manufacturer’s protocol. RNA quantity and purity was measured using NanoDrop 2000c spectrophotometer (Thermo-scientific, Wilmington, USA). cDNA synthesis was performed using M-MLV Reverse Transcriptase from 1 μl of total RNA according to the manufacturer’s protocol (Promega, Madison, USA). Analysis of mRNA expression by reverse transcription RT-PCR was carried out using standard protocols. The following RT-PCR cycle profile was used: 10 min at 95 °C, followed by 40 cycles of 15 s at 95 °C for denaturation, 20 s at 60 °C for combined annealing, and 10 s at 72 °C for extension. Real Time PCR was performed using the custom primers (Invitrogen, Carlsbad, USA) for β-actin (forward 5′-AGGAGTACGATGAGTCCGGCCC-3′ and reverse 5′-GCAGCTCAGTAACAGTCCGCCT-3′, accession number NM 031144.2) as housekeeping and TGF-β 1 (forward 5′-CAACCCGGGTGCTTCCGCAT-3′ and reverse 5′-TGCTCCACCTTGGGCTTGCG-3′, accession number NM 021578.2) as target gene.

### IDO activity

Renal IDO activity was accessed by detection of kynurenine after digestion of tryptophan by IDO presents in the renal tissue. The method used was adapted using two methods previously described [23, 24]. Briefly, renal tissue was homogenized in potassium phosphate buffer (50 mM, pH 6.0) using a hand held homogenizer (D 130, Wiggen Hauser,Berlin, Germany), and then the homogenate was centrifuged at 4 °C (5 min, 12,000 g). In parallel, a standard curve was constructed with the following concentrations: 0.5 μM, 1.0 μM, 2.0 μM, 4.0 μM, 8.0 μM, and 16.0 μM. The supernatant (or standard) (100 μl) was mixed with 100 μl of digestion buffer (500 mM-potassium phosphate, 20 mM-ascorbic acid, 200 μg/ml-catalase, 10 nM methylene blue, 400 μM-L-tryptophan). The mixing was incubated at 37 °C for 60 min, and then desproteinized by adding 30% trichloroacetic acid (5:1, *v*/v), followed by incubation at 65 °C for 15 min. The samples were centrifuged at 11,500 g for 15 min, and the supernatants (100 μl) were added to 4-(dimethylamino) benzaldehyde (2% in acetic acid), and read by spectrometry at 480 nm.

### MDCK cell culture

MDCK cells (Madin-Darby Canine Kidney, NBL2; American Type Culture Collection-ATCC, Manassas, VA, USA) were acquired and cultured in Dulbecco’s Modified Eagle’s Medium (DMEM; Vitrocell, Campinas, Brazil) supplemented with 10% fetal bovine serum (FBS) and penicillin-streptomycin (Sigma-Aldrich, St. Louis, MO) and maintained at 37 °C with 5% CO_2_.

To analyze the effect of TGF-β1 on IDO expression, MDCK cells were seeded in 24-well plates (3X10^4^ cells per well). The cells were incubated with 1 ng/ml of TGF-β1 (R&D Systems Inc., Minneapolis, MN) in DMEM 1% FBS for 48 h. MDCK in DMEM 1% FBS without TGF-β1 was used as control. To promote IDO inhibition, we used DMEM containing 1 mM 1-methyl-D-tryptophan (MT; cat 452,483, Sigma-Aldrich, St. Louis, MO). All experiments were performed in triplicate.

### Immunofluorescence and immunocytochemistry

IDO was analyzed by immunofluorescence. MDCK cells were fixed in 4% paraformaldehyde for 15 min at 37 °C and then maintained in PBS containing 0.5% bovine serum albumin and 0.1% triton X-100. As primary antibody, monoclonal mouse PE-conjugated anti-IDO was used (1:25; Clone#700838, R&D Systems Inc., Minneapolis, MN), and incubation for 2 h at 37 °C was carried-out. Fluorescence immunostaining was detected using Zoe™ Fluorescent Cell Imager (Bio-Rad Laboratories, Hercules, CA).

Immunocytochemistry was carried-out to analyze the αSMA expression in MDCK cells. The cells were smoothly washed using PBS, and then fixated for 10 min into a 4% paraphormaldehyde solution. For the endogenous peroxidase blocking step, we prepared a 3% H_2_O_2_ solution (in methanol) and incubated the cells for 30 min, covered from light. The cells were incubated with anti-αSMA (IS700; Dako Co, Denmark) at 4 °C for 12 h. To complete the sandwich, the cells were incubated with LSAB+ System-HRP reagents (K0690; Dako Co, Denmark). Finally, DAB substrate-chromogen was used to complete the reaction (K346811; Dako Co, Denmark).

### Kynurenine measurement

High-Performance Liquid Chromatograph (HPLC) was performed to measure kynurenine in the supernatants of the TGF-β 1-stimulated MDCK cells and of the unstimulated MDCK cells (control).

Supernatants were deproteinized by centrifugation at 5000 g (15 min at 4 °C) with 10% trichloroacetic acid (1:1, *v*/v), filtered in millipore 0.25 μm and 20 μL was injected into HPLC instrument equipped with UV detector (YL-9300; YL Instrument, Anyang, Korea). Data were obtained using a reversed phase column (LUNA RP-18, 25 cm × 4.5 mm; Phenomenex, Torrance, Ca, US), at room temperature. Separation was done in the following mobile phase: buffer sodium acetate 10 mM in MilliQ water (A) and acetonitrile (B): 0–1 min (20% B); 1.01–1.5 min (5% B); 1.51–8 min (4% B). The flow rate was kept constant at 1 mL/min and peaks were detected at 254 nm. All chemicals used in the analysis, such as acetonitrile and acetate buffer, were of HPLC grades and were purchased from Sigma and Merck.

A kynurenine standard curve was constructed (2.0 μM, 4.0 μM, 8.0 μM, and 16.0 μM). Injections were done in triplicate and kynurenine was detected by 254 nm UV. Linearity was observed in the concentration range 0.5 to 100 μM of kynurenine and the samples were quantified against the calibration standard curves, where y is the peak in Voltage (mV) and x the concentration in μM (y = 1.1×-0.0468 R2 = 0.998) and retention times of 2.1.

### Scratch-wound migration

MDCK cells were seeded in 24-well plates (3X10^4^ per well) and cultured until reaching 80% confluence (approximately 24 h). One scratch per well was carried out using a 10 μl pipette tip and four images per well were taken at 40X magnification under an inverted microscope (Ti-S; Nikon Corp., Tokyo, Japan). After 12 h, additional images were acquired. Each scratch-wound area was calculated using the ImageProPlus 6.0 program (Media Cybernetics Inc., Bethesda, MD).

### Statistical analysis

Data are presented as the mean ± SEM. For parametric data, one-way analysis of variance with pairwise comparisons was conducted according to the Newman-Keuls formulation. For non-parametric data, Kruskal-Wallis or Wilcoxon was applied. A *p*-value less than 0.05 was considered significant. The data were analyzed using the SPSS software (version 23.0, SPSS Inc., Chicago, IL, USA).

## Results

### Renal morphology and macrophages

Seven days after ureteral ligation, Masson’s Trichrome-stained sections revealed that obstructed kidneys presented tubular dilatation accompanied by tubular atrophy, and a significant expansion of interstitial area with accumulation of collagen and cell infiltrates. Glomeruli and vessels were preserved. No morphology changes were observed in CL kidneys. Quantitative analysis showed a significant increase of the interstitial area of the obstruct kidney compared to SHAM and CL kidneys (Table 1).

**Table 1:** Characterization of EMT in the UUO model

		SHAM	CL	UUO
Renal fibrosis	Interstitial area (%)	0.3 ± 0.1	0.9 ± 0.1	13.4 ± 2.6^‡^
Macrophages	Interstitial area (cells/field)	6.2 ± 0.8	16.0 ± 2.7	75.2 ± 12.6^‡^
E-cadherin	Tubular % positive tubules	20.8 ± 5.4	21.1 ± 11.6	02.7 ± 1.1
αSMA	Tubular % positive cells	27.2 ± 6.5	35.8 ± 0.90	61.4 ± 4.2^†^
	Interstitial positive cells/field	17.7 ± 7.1	74.5 ± 23.5	368.8 ± 45.8^‡^
Vimentin	Tubular % positive cells	02.0 ± 0.5	02.5 ± 0.50	033.8 ± 2.7^‡^
	Interstitial positive cells/field	13.0 ± 0.9	17.0 ± 1.20	141.3 ± 8.8^‡^
TGF-β 1	Relative expression	1.0 ± 2.2	6.8 ± 0.3	14.7 ± 0.1^‡^

UUO kidneys presented increase of interstitial fibrosis, macrophage infiltrating, markers of mesenchymal cells, and TGF-β1 expression. Renal fibrosis was evaluated by Masson’s trichrome-stained interstitial area. Interstitial macrophages were identified by immunohistochemistry. EMT markers were also analyzed by immunohistochemistry. Real-time PCR was used to analyze TGF-β1 expression in the renal tissue. Data are expressed as the mean ± SEM. ^†^ *p* < 0.001 versus SHAM and CL; ^‡^ *p* < 0.0001 versus SHAM and CL

Immunohistochemistry analysis showed that the mean number of macrophages was significantly higher in obstructed kidney compared to SHAM and CL kidneys (Table 1). In particular, macrophages infiltration was predominantly in the interstitium.

### EMT in UUO

**Fig. 1:**
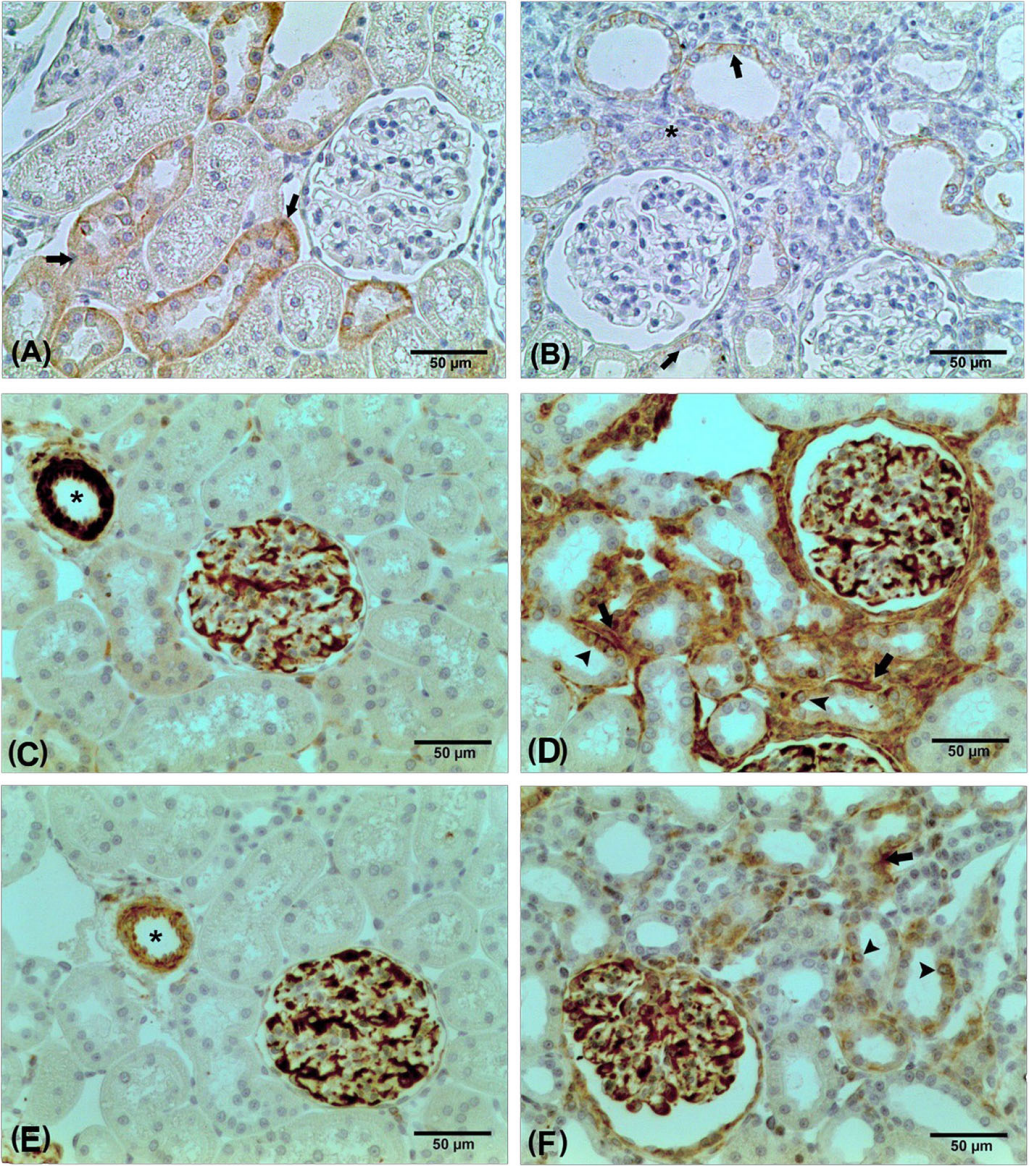
Representative images of immunohistochemistry for E-cadherin (A and B), αSMA (C and D), and vimentin (E and F). E-cadherin staining was remarkable in tubules of CL kidneys (**a**), while it was significantly reduced in obstructed kidneys (**b**). Arrows in A and B indicate E-cadherin staining in tubules, and the asterisk indicates the interstitial cells. Immunohistochemistry for αSMA revealed that in CL kidneys (**c**), αSMA staining was found constitutively in vessels (asterisk) and within glomeruli (mesangial cells). In obstructed kidneys (**d**), the αSMA staining was robust in interstitium (arrow) and in tubules (arrowhead). Similar to αSMA, vimentin staining was found in vessels and glomeruli of the CL kidneys (**e**), while obstructed kidneys (F) presented additional positivity for vimentin in interstitial cells (arrow) and in tubules (arrowhead)

To characterize the EMT, immunostaining for E-cad, αSMA and vimentin was carried-out. While E-cad, an epithelial marker, was constitutively expressed in tubules of the SHAM and CL kidneys, it was drastically reduced in obstructed kidneys, especially in areas of interstitial expansion (Table 1, Fig. 1). In contrast, a moderate immunostaining for αSMA and vimentin, two mesenchymal markers, was found in tubular cells of the SHAM and CL kidneys, while a significant expression was found in tubular cells of the obstructed kidneys (Table 1, Fig. 1). Parallel, αSMA^+^ and vimentin^+^ interstitial cells were rarely in SHAM and CL kidneys, but a robust number was found in obstructed kidneys (Table 1, Fig. 1). CL kidneys constitutively expressed α-SMA in smooth muscle cells of the arterioles and thus normal expression was not influenced by ureteral obstruction.

### TGF-β 1 expression in UUO model

**Fig. 2:**
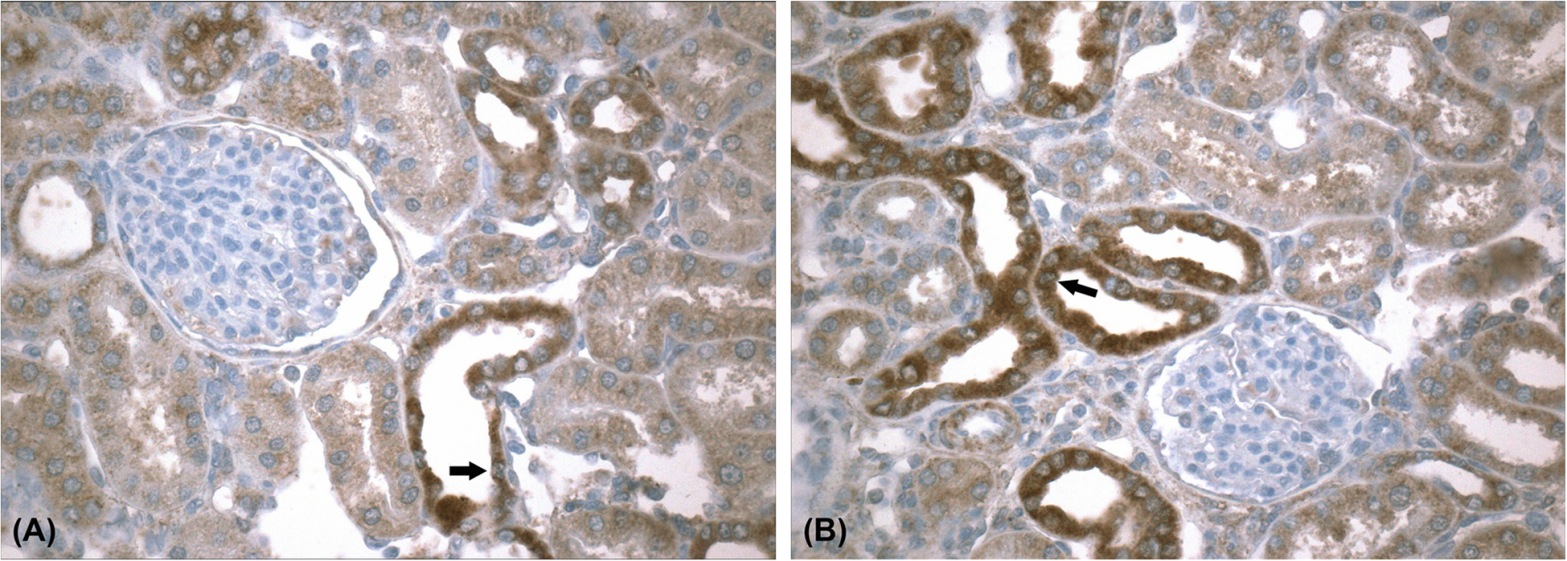
Immunohistochemistry for TGF-β 1 in CL kidney (**a**) and obstructed kidney (**b**). TGF-β 1 immunostaining was found predominantly in distal tubules (arrow), being more pronounced in obstructed kidneys

To further confirm the mechanisms involved in the pathogenesis of renal fibrosis, we measured TGF-β 1 in kidney tissue by immunohistochemistry and real-time PCR. As shown in Fig. 2, TGF-β 1 expression was higher on UUO kidneys. In real-time PCR (Table 1), UUO also promoted a significant increase in TGF-β 1 expression than SHAM kidneys. Curiously, CL kidney presented higher expression of TGF-β 1 compared to SHAM kidneys. These data confirmed the association between fibrosis formation and TGF-β 1 expression in this model.

### Immunostaining for IDO in renal tissue

**Fig. 3:**
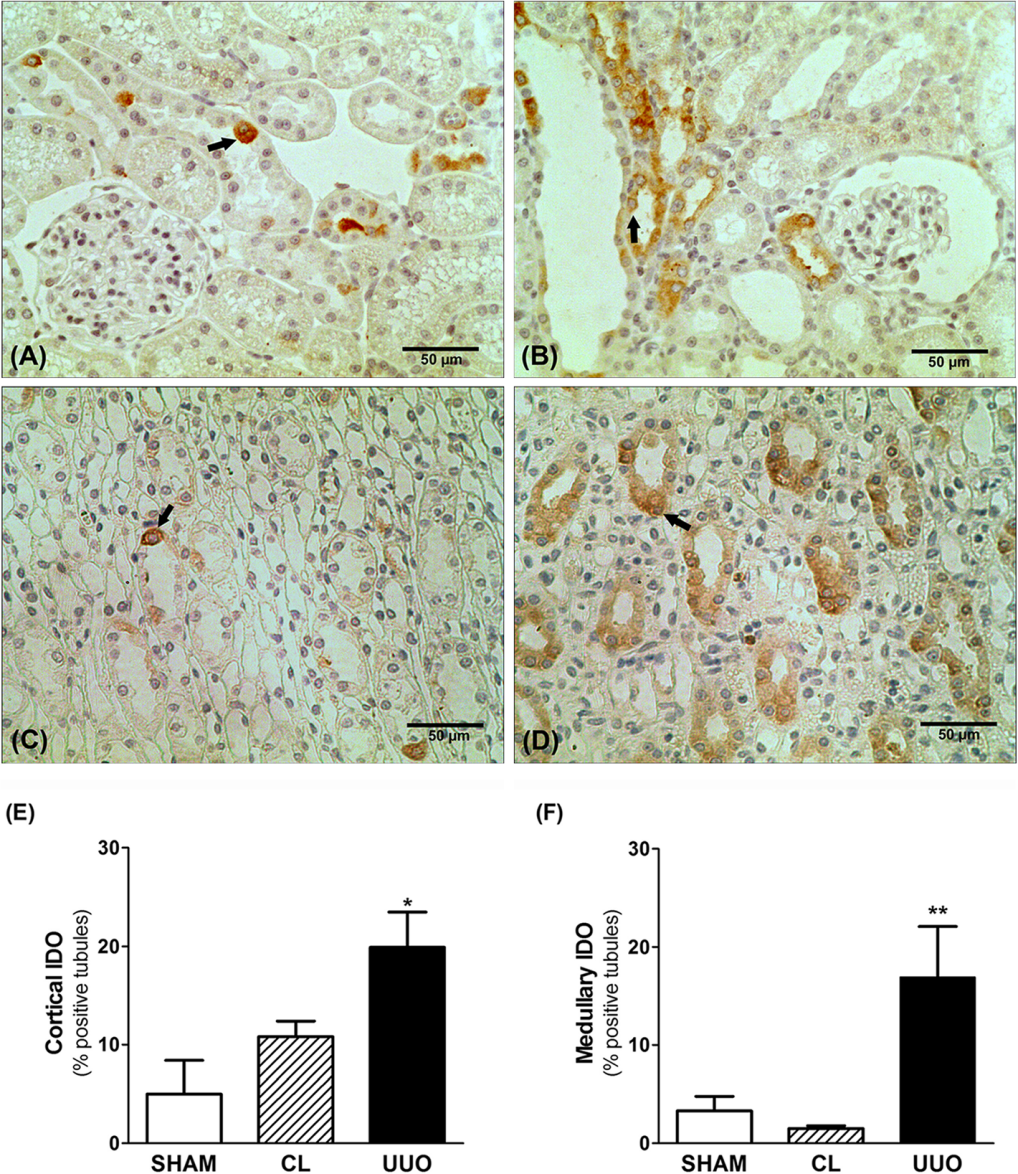
Immunohistochemistry for IDO in renal cortex (**a** and **b**) and renal medulla (**c** and **d**) of the CL kidney (**a** and **c**) and obstructed kidney (**b** and **d**). IDO staining was found in tubular cells (arrow). IDO staining was significant higher in UUO kidneys in both cortex (**e**) and medulla (**f**) (*n* = 5). **p < 0.01 vs. SHAM and CL; *p < 0.05 vs. SHAM and CL

As illustrated in Fig. 3, CL kidneys presented IDO immunostaining in few cortical and medullary tubules (similar to SHAM). In UOO kidneys, the immunostaining for IDO was significantly increased in both areas (Fig. 3). The quantitative analysis showed that the percentage of IDO^+^ cortical tubules was significantly higher in UUO kidneys than in SHAM and CL kidneys (19.9 ± 3.6% in UUO versus 5.0 ± 3.4% in SHAM and 10.8 ± 1.6% in CL, *p* < 0.05), and similar effect was found in renal medulla (16.9 ± 5.2% in UUO versus 3.3 ± 1.5% in SHAM and 1.5 ± 0.3% in CL, *p* < 0.05) (Fig. 3).

### IDO activity in UUO model

**Fig. 4:**
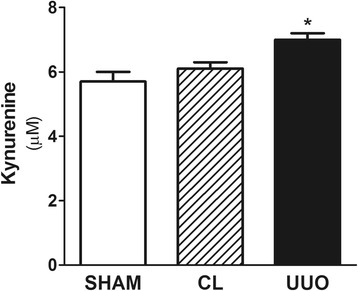
IDO activity in renal tissue accessed by kynurenine measurement (*n* = 5). IDO activity was significantly higher in UUO. *p < 0.05 vs. SHAM and CL

In order to analyze IDO activity in the renal tissue, we measured kynurenine after digestion of tryptophan by renal IDO. IDO activity was significantly higher in UUO kidneys compared to SHAM and CL kidneys (7.0 ± 0.2 μM in UUO versus 5.7 ± 0.3 μM in SHAM and 6.1 ± 0.2 μM in CL, *p* < 0.05) (Fig. 4).

### Effect of TGF-β 1 on IDO expression in MDCK cells

**Fig. 5:**
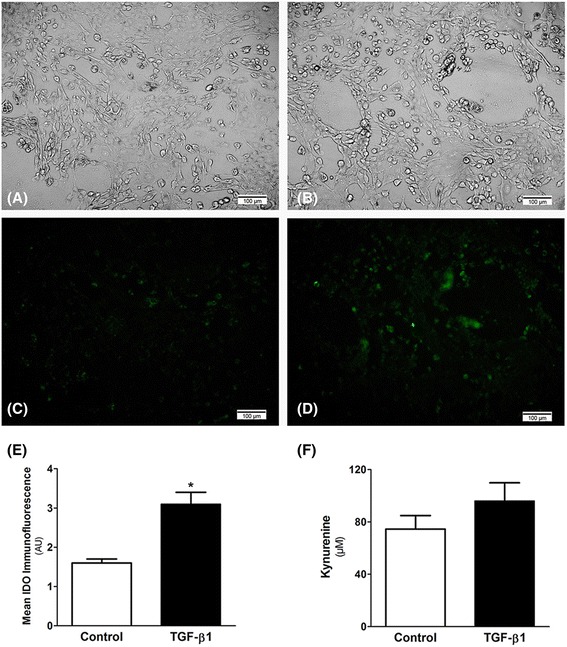
MDCK cells were cultured in DMEM medium supplemented with 10% FBS at 37 °C with 5% CO_2_. MDCK cells were stimulated with TGF-β 1 (1 ng/ml) for 48 h (**b** and **d**) and unstimulated cells were used as control (**a** and **c**). Cells before the expose to fluorescence (**a** and **b**). Immunofluorescence for IDO (**c** and **d**) in MDCK cells. The immunofluorescence for IDO was significantly higher in TGF-β 1-stimulated cells (**e**). HPLC measurements demonstrated that the concentration of kynurenine, the main IDO catabolite, was increased in the supernatant of TGF-β 1-stimulated cells, but no statistical significance was found (**f**). Triplicate for each condition were performed. *p < 0.05 vs. Control

As demonstrated in Fig. 5, TGF-β 1 increased the immunofluorescence staining for IDO in MDCK cells after 48 h (1.6 ± 0.1 arbitrary unit in control versus 3.1 ± 0.3 arbitrary unit in TGF-β 1-stimulated cells; *p* < 0.05). Although the kynurenine was increased in the supernatant of the TGF-β 1-stimulated cells, no statistical significance was observed (7.5 ± 1.0 μM in control versus 9.6 ± 1.4 μM in TGF-β 1-stimulated cells).

### Effect of IDO inhibition on EMT in MDCK cells

**Fig. 6:**
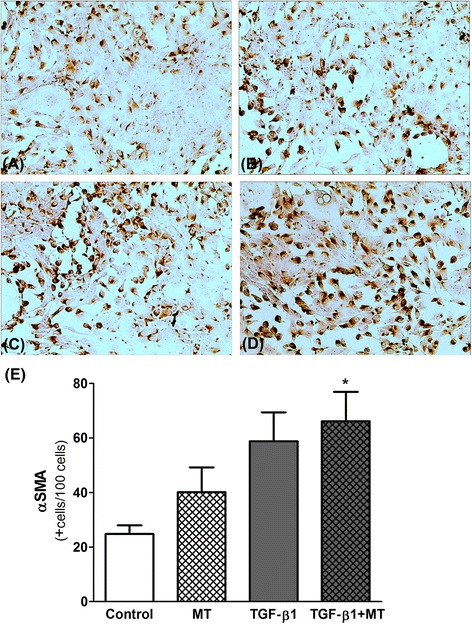
Immunocytochemistry for αSMA in MDCK cells. MDCK cells were cultured in DMEM medium supplemented with 10% FBS at 37 °C with 5% CO_2_. **a** Unstimulated MDCK cells (Control), (**b**) MDCK cells incubated with the IDO inhibitor 1-methyl-D-tryptophan (MT; 1 mM), (**c**) TGF-β 1-stimulated MDCK cells, and (**d**) TGF-β 1-stimulated MDCK cells treated with MT. The quantitative analysis demonstrated that the number of αSMA^+^ cells was increased by TGF-β 1 and the treatment with MT potentiated this effect (**e**). The conditions were performed in triplicate. *p < 0.05 vs. Control

αSMA expression was analyzed by immunocytochemistry and used to identify a mesenchymal phenotype for TGF-β 1-stimulated MDCK cells, reflecting the EMT phenomenon. As demonstrated in Fig. 6, TGF-β 1 increased the αSMA expression in MDCK cells, and treatment with MT potentiated this effect (24.8 ± 3.2 αSMA^+^ cells % in control, 40.1 ± 9.1 αSMA^+^ cells % in MT, 58.8 ± 10.6 αSMA^+^ cells % in TGF-β 1, and 66.1 ± 10.8 αSMA^+^ cells % in TGF-β 1 + MT; *p* < 0.05 control versus TGF-β 1 + MT). MT alone increased the number of αSMA^+^ cells when compared to control, but no statistical significance was found.

**Fig. 7:**
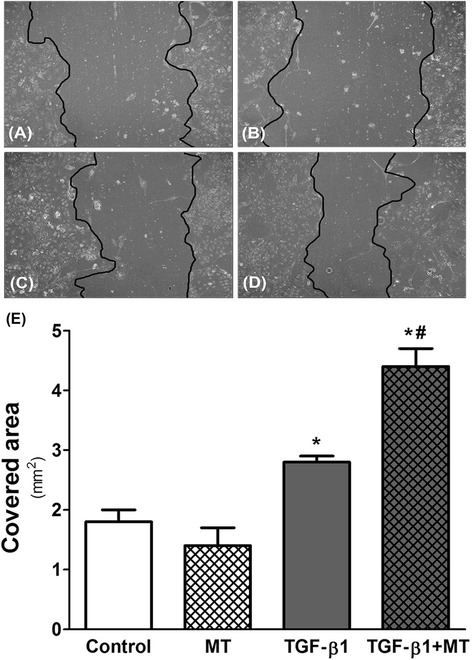
Scratch-wound migration of the MDCK cells. MDCK cells were seeded in 24-well plates and one scratch per well was carried out using a 10 μl pipette tip. After 11 hrs of migration, images were acquired. **a** Unstimulated MDCK cells (Control), (**b**) MDCK cells incubated with the IDO inhibitor 1-methyl-D-tryptophan (MT; 1 mM), (**c**) TGF-β 1-stimulated MDCK cells, and (**d**) TGF-β 1-stimulated MDCK cells treated with MT. TGF-β 1 promoted cell migration, and treatment with MT potentiated this effect (**e**). *p < 0.05 vs. Control and MT; ^#^ *p* < 0.05 vs. TGF-β 1

Additionally to the αSMA expression, we investigated the migratory capacity of the MDCK cells. The Fig. 7 illustrates the area covered by MDCK cells. TGF-β 1 potentiates the migratory capacity of the cells, and the MT treatment significantly intensified this effect (1.8 ± 0.2 mm^2^ in control, 1.4 ± 0.3 mm^2^ in MT, 2.8 ± 0.1 mm^2^ in TGF-β 1, and 4.4 ± 0.3 mm^2^ in TGF-β 1 + MT; *p* < 0.05 control versus TGF-β 1 and versus TGF-β 1 + MT).

## Discussion

IDO has been correlated with some types of renal disease. However, its correlation with renal fibrosis had not been explored yet. Here, we demonstrated that IDO increases during UUO, a model of nonimmune-mediated renal disease with fibrogenesis.

UUO model is characterized by robust renal inflammation, involving a complex sequence of events wherein mesenchymal fibroblasts become “activated” and culminate in production and deposition of extracellular matrix. Analysis and measurement of histological samples revealed renal damage, characterized by remarkable tubulointerstitial alterations, including tubular dilatation and atrophy, as well as a remarkable fibrosis in the interstitium. These morphological changes were described previously [25]. In our study, immunohistochemical experiments identified an increased number of macrophage, accompanied by increased tubulointerstitial expression of mesenchymal markers as well as αSMA and vimentin and by the loss of the epithelial marker e-cadherin in the tubules of obstructed kidneys. These findings are in agreement with previous observations [25–28].

Using real-time PCR, we demonstrated that TGF-β 1 was overexpressed in obstructed kidneys, and the immunostaining experiments shown that TGF-β 1 was predominantly expressed in cells of the distal tubules. Even though we can observe a background in the immunohistochemistry for TGF-β 1, the results matched the qRT-PCT findings and are in agreement with previous data [29] and support the hypothesis that fibrosis is typically the end result of chronic inflammatory reactions, induced by a variety of stimuli including tissue damage and cytokines such as TGF- β 1 [28, 29].

In this study, we demonstrated that tubular cells constitutively express IDO, preferably in distal tubules of the nephrons. The renal expression of IDO was significantly increased in rats underwent to UUO. In addition to the immunostaining experiments, we accessed the IDO activity. Obstructed kidney presented a higher IDO activity when compared to the contralateral kidney. Others described the tubular expression of IDO. In biopsies from patients with kidney transplantation, Brandacher et al. shown that IDO expression was significantly increased in tubular epithelium of rejected kidneys when compared with nonrejected allograft [15]. Mohib et al. demonstrated that mice underwent to renal ischemia-reperfusion injury presented abundant amounts of IDO in the tubular epithelium without distinction of the type of tubular cells [19]. In a model of type 2 diabetic nephropathy, IDO was found in interstitial cells, in association with pro-inflammatory cytokines [20]. In adriamycin-induced renal failure mice model, IDO was found in dilated tubules, correlating with worsening of disease [30]. The mechanism to explain why IDO is overexpressed in tubular cells during renal fibrogenesis remains unclear. Paralleling with the female reproductive system, the EMT program is fundamental to the embryo implantation, embryogenesis, and organ development [10]. Curiously, in the first trimester of pregnancy, IDO has been found in decidualization area, in which syncytiotrophoblast, cytotrophoblast and invasive extravillous trophoblast make intensively EMT [20]. Because EMT is an essential phenomenon for renal fibrogenesis, we hypothesized that IDO could influence tubular EMT.

Our results showed that IDO accompanied the renal fibrosis, and its expression coincides locally with TGF-β 1. To understand the possible mechanism for linking IDO with TGF-β 1-induced renal fibrosis, we used the MDCK cells, a representative lineage for the distal tubular cells, and focused in the EMT program. When MDCK cells were incubated with TGF-β 1, IDO was overexpressed, and kynurenine in the supernatant was increased. The treatment of these TGF-β 1-stimulated cells with MT exacerbated the cytoplasmic αSMA immunostaining and intensified the migratory capacity. It is possible that IDO mediates EMT in tubular cells. Pallota et al. demonstrated that IDO is regulated by TGF-β 1 in dendritic cells, stablishing an important pathway to promote differentiation for acquiring regulatory phenotype [11].

The mechanism by which IDO acts in tubular cells is still unclear. However, the results of this study lead us to speculate that IDO has a renoprotective property since its inhibition potentiated tubular EMT. A possible mechanism could be via activation of a stress response dependent on the eIF2α kinase general control nonderepressible 2 (GCN2). The tryptophan deprivation promoted by IDO in the microenvironment deflagrates the GCN2 activation, which can trigger mechanisms of renoprotetion. Reinforcing this theory, the use of the halofuginone, a GCN2 activator, prevents extracellular matrix deposition in a murine model of diabetic nephropathy through downregulation of TGF-β signaling and oxidative stress [31]. In the same sense, Eleftheriadis et al. demonstrated that tryptophanol, another GCN2 activator, had protective effect on endothelial cells by preventing the high-glucose-induced injury [32].Working with a mouse nephrotoxic serum nephritis model, Chaudhary et al. demonstrated that the activation of GCN2 driven by IDO was effective in suppressing renal injury by inducing autophagy [33]. Interestingly, renal medullary cells that are regularly exposed to high osmolality stress (as part of the renal physiology) are protected by activation of the GCN2 pathway [34]. Loss of GNC2 decreases cell survival and induces the expression of activated caspase-3 [34].

Another pathway mediated by IDO-promoted tryptophan deprivation is the mTOR [35]. IDO activity inhibits the tryptophan sufficiency signal that stimulates mTOR, an important pathway to induce EMT and consequently renal fibrosis [36]. It is possible that the deleterious effect that we found with MT treatment inducing EMT in MDCK cells is related to the capacity of MT acts like tryptophan for the inhibition of CGN2 and/or for the activation of the mTOR pathway. In fact, the activation of mTOR seems to be strongly related to many renal diseases, playing an important role in diabetic neuropathy, acute kidney injury, polycystic kidney disease, glomerulopathy, intrarenal inflammation, and interstitial fibrosis [37]. Although mTORC1 sensitivity to IDO-induced L-tryptophan depletion is not found in every human cell types like occurs in T-cells [38, 39], in the renal cells this effect was not yet demonstrated.

Lastly, another action of IDO may be triggered by activation of the aryl-hydrocarbon receptor (AhR) via the kynurerine pathway. Besides the AhR acting directly controlling the expression of specific genes, it acts by inhibiting the hypoxia-induced factor (HIF) by antagonizing with HIF-1α subunit [40, 41]. Because the prolonged activation of HIF signaling in renal epithelial cells leads to renal fibrosis [42], it is possible that IDO has a renoprotective effect also by activating AhR.

Based on these observations, further studies evaluating the connection of IDO with these pathways may be interesting to understand more deeply the action not only of IDO but its related molecules in the development of renal injuries.

## Conclusion

IDO is constitutively expressed in tubular cells and increases during renal fibrogenesis. Although IDO expression and activity are potentiated by TGF-β 1, IDO does not act intensifying the renal fibrosis. In contrast, IDO had a renoprotective effect, since its inhibition potentiated the TGF-β 1-induced EMT. Similarly as in the inflammatory process, in which IDO is a prominent molecule that avoids potentially harmful inflammatory responses, it is likely that IDO may have a relevant physiopathological role by softening the renal fibrosis during the overexpression of TGF-β 1. This balance between IDO and TGF-β 1 should be considered when developing therapeutic interventions on the basis of IDO modulation.

## Abbreviations

CKD: Chronic kidney disease
IDO: Indoleamine 2, 3-dioxigenase
MT: 1-methyl-D-tryptophan
UUO: Unilateral ureteral obstruction
